# A hands-free wearable electrolarynx for communication in tracheostomized mechanically ventilated critically ill patients: a case series

**DOI:** 10.1007/s00540-025-03481-2

**Published:** 2025-03-12

**Authors:** Koji Sato, Junji Genda, Seiki Deguchi, Takumi Taniguchi

**Affiliations:** 1 Intensive Care Unit, Kanazawa University Hospital, 13-1 Takara-machi, Kanazawa, 920-8641 Japan; 2 Department of Rehabilitation, Kanazawa University Hospital, 13-1 Takara-machi, Kanazawa, 920-8641 Japan

**Keywords:** Communication tools, Electrolarynx, Tracheostomy, Intensive care, Mechanical ventilation

## Abstract

The electrolarynx facilitates communication for tracheostomized mechanically ventilated patients. However, its effectiveness is often constrained by patient weakness. A hands-free electrolarynx offers a potential solution, enabling speech without the need to hold the device. This case series evaluated speech intelligibility with a hands-free electrolarynx in tracheostomized, mechanically ventilated patients. Seven tracheostomized patients were enrolled and instructed to use both the conventional and hands-free electrolarynxes. Speech intelligibility was assessed using the 5-point electrolarynx effectivity score (EES). Patients who scored ≥4 were defined as effective users. The median EES (interquartile range [IQR]) for the hands-free electrolarynx was 3.5 (2.5–4), with three effective users (42.9%). The median EES for the conventional electrolarynx when operated by the research staff was 4 (IQR: 3–4), with four effective users (57.1%), indicating no significant difference compared with the hands-free electrolarynx (p=0.7). However, none of the patients were able to effectively and independently utilize the conventional device, leading to a significantly lower median EES of 2 (IQR: 2–2.6; p = 0.03). The hands-free electrolarynx achieves greater intelligibility than the conventional electrolarynx when used independently by patients. The hands-free electrolarynx has the potential to minimize the need for external assistance and enhance autonomous communication.

**Supplementary Information:**

The online version contains supplementary material available at 10.1007/s00540-025-03481-2.

## Introduction

The electrolarynx is a battery-powered handheld device that is pressed onto the skin of the neck to transmit the vibrated electronic sound into the oropharyngeal cavity. Speech is produced by moving the articulators, including the lips, tongue, and jaw. The device was initially developed to assist patients following laryngectomy [1] and has also been utilized in tracheostomized, mechanically ventilated patients [2, 3]. By facilitating verbal communication, it may also help alleviate anxiety in this patient population [4]. During phonation, the electrolarynx is positioned externally against the neck by the speaker or an assistant. Although the electrolarynx can alleviate anxiety, critically ill patients often face additional challenges, including insufficient muscle strength to hold the device steadily and apply appropriate pressure, as well as limited ability to undergo the necessary training, particularly in identifying the optimal device position and coordinating mouth movements. [4]. A hands-free wearable electrolarynx has been developed, allowing communication without the need to hold or reposition the device [5]. This device possesses two notable features: (1) it enables speech without being hand-held, as it wraps around the neck to ensure more stable voice production, and (2) it includes an easy-to-operate on/off switch designed to assist patients with limited muscle strength. These features may overcome the barriers that have previously hindered the use of the electrolarynx in patients requiring mechanical ventilation.

This case series evaluated the feasibility and speech intelligibility associated with using a hands-free electrolarynx in tracheostomized, mechanically ventilated patients. We hypothesized that the hands-free electrolarynx might enable patients to produce a more unmistakable artificial voice independently than the conventional electrolarynx.

## Methods

A consecutive case series of seven tracheostomized patients was conducted between Jury 2022 and November 2023. This study was conducted with the approval of the Ethical Committee of Kanazawa University (approval no: 113803-1). Written informed consent was obtained from each participant. The study protocol was registered with the University Hospital Medical Information Network Clinical Trials Registry (UMIN-CTR: UMIN000048039). The inclusion criteria for this study were as follows: Japanese patients aged ≥20 years who were receiving mechanical ventilation and had undergone a tracheostomy in the intensive care unit (ICU); were expected to have difficulty weaning early from ventilation; were capable of communication; and provided informed consent to participate. We excluded patients with comorbidities of the central nervous system; those who had undergone maxillofacial or head and neck surgery with surgical wounds or anatomical abnormalities in the neck or oral cavity that could impede device placement; individuals with cognitive impairment, delirium, or agitation that made device placement difficult; and patients with very severe frailty or depressive symptoms who were deemed unsuitable for the study by the research staff.

### Study procedures

**Fig. 1:**
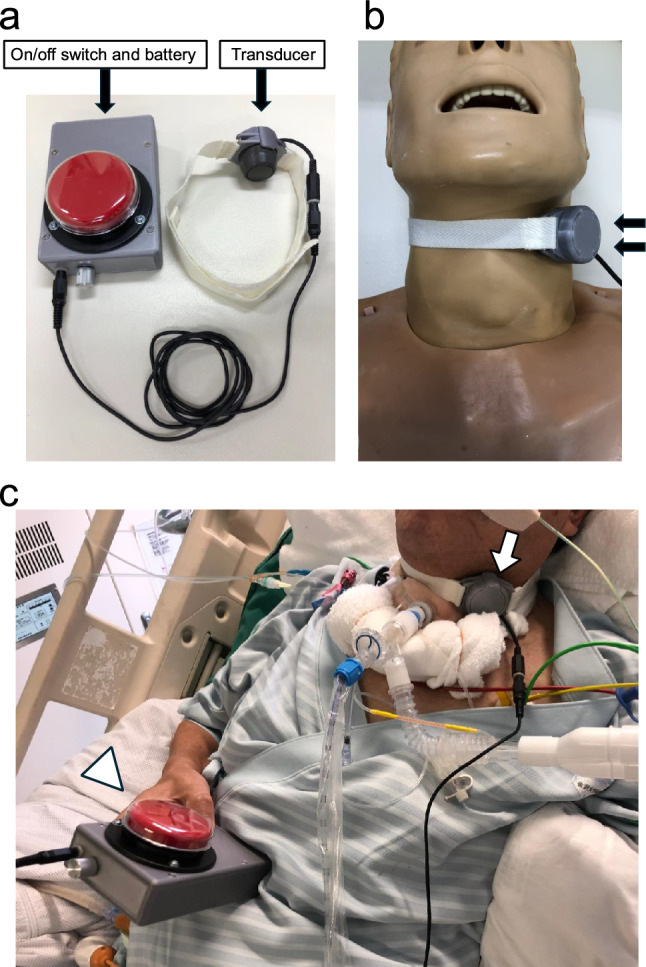
The hands-free electrolarynx. Figures 1a and 1b show the structure of the hands-free electrolarynx. The device has a cylindrical transducer that vibrates the throat to produce sound and an easy-to-use on/off switch and battery. Figure 1c shows a tracheostomized, mechanically ventilated patient using the device for speech. The white arrow indicates the transducer attached to the patient’s neck, while the white arrowhead points to the on/off switch operated by the patient

Speech intelligibility was assessed after a brief orientation. Initially, speech produced using a conventional electrolarynx (NU-VOIS II; Nu-Vois, LLC) was evaluated, followed by a hands-free electrolarynx (provided by Masaki Takeuchi, Graduate School of Electricity Engineering, The University of Tokyo, Tokyo, Japan) (Figure 1). For the hands-free electrolarynx, the participant only operated the on-off switch (Supplementary file 1; Online Resource 1). The effectiveness of electrolarynx speech was assessed by two independent raters and graded using the 5-point Electrolarynx Effectivity Score (EES) developed by Tuinman et al. (1 = no improvement in intelligibility; 5 = very effective, capable of producing sentences) (Supplementary file 2; Online Resource 2) [6]. Their scores were averaged to yield a final EES. Patients with an EES of ≥4 were classified as effective users. The primary outcomes were the EES for both the hands-free and conventional electrolarynx, and the number of effective users for each device. Safety outcomes were also assessed by monitoring adverse respiratory and hemodynamic events, including reductions in oxygen saturation from baseline and life-threatening arrhythmias during electrolarynx use. Additionally, we evaluated whether the device could interfere with tracheostomy tubes and recorded any other device-related complications.

### Statistical analysis

All statistical analyses were conducted using STATA, version 15.1 (StataCorp, College Station, TX, USA). Continuous variables are presented as median (interquartile range, IQR) and categorical variables as frequencies and percentages. Inter-group comparisons were performed using the Wilcoxon signed-rank test. Statistical significance was set at p<0.05.

## Results

Seven tracheostomized patients were enrolled, achieving a consent rate of 38.9% (Supplementary file 2; Online Resource 3). Most participants were male (male: n = 6, 85.7%; female: n = 1, 14.3%), with four (57.1%) admitted for respiratory diseases. The median age was 69 years (IQR 63–71 years). Regarding ventilation modes, six patients (85.7%) were managed with pressure support, while one was treated with pressure control. The median mechanical ventilation duration was 13 days (IQR 8–23 days) (Table 1). All patients were maintained at a Richmond Agitation-Sedation Scale of 0 throughout the assessment, and details of the sedative and narcotic drugs administered are provided in Online Resource 4 (Supplementary file 2).

**Table 1:** Demographic data

Case	Age (years)	Sex	Charlson comorbidity index	Primary diagnosis	SOFA score	Ventilator mode	PEEP (cmH_2_O)	P/F ratio	Ventilation duration (days)
1	79	F	7	Cardiomyopathy	7	PS	5	400	23
2	69	M	4	Pneumonia	2	PS	5	304	13
3	66	M	6	Empyema	2	PS	8	240	9
4	63	M	6	Pneumonia	4	PS	7	89	15
5	47	M	0	Acute myocarditis	2	PS	5	260	43
6	69	M	2	Spinal cord injury	4	PCV	8	170	4
7	71	M	4	Alveolar hemorrhage	5	PS	7	218	8
Median (IQR)	69 (63–71)		4 (2–6)		4 (2–5)		7 (5–8)	240 (170–280)	13 (8–23)

*F* female, *M* male, *PCV* pressure control mode, *PEEP* positive end-expiratory pressure, *P/F* partial pressure of oxygen in arterial blood to the fraction of inspiratory oxygen concentration, *PS*, pressure support mode, *IQR* interquartile range, *SOFA* sequential organ failure assessment

The median EES for the hands-free electrolarynx was 3.5 (IQR 2.5–4) (Table 2). One patient achieved the maximum EES of 5. Additionally, three out of seven patients (42.9%) were classified as effective users, with EES above 4 when using the hands-free electrolarynx. Only one patient (Case 6) with tetraplegia due to spinal cord injury required assistance to operate the on/off switch. When a research team member operated the conventional electrolarynx, the median EES was 4 (IQR 3–4), showing no significant difference compared with the hands-free electrolarynx (p = 0.7). Four patients (57.1%) were considered effective users. However, none of the patients were classified as effective users when they used the device independently; the median EES dropped to 2 (IQR 2–2.6), significantly lower than the hands-free electrolarynx (p = 0.03).

**Table 2.** Five-point electrolarynx effectivity scores for the hands-free and conventional electrolarynx devices

Case	Hands-free electrolarynx	Conventional electrolarynx		
		Operated by research staff	Without assistance	
1	2.5	3		2
2	5	4		3
3	4	4		2
4	2	2		2
5	3.5	4		2
6	3*	3		N
7	4	4		2.5
Median (IQR)	3.5 (2.5–4)	4 (3–4)		2 (2–2.6)

5-point electrolarynx effectivity score: 1 = no improvement in intelligibility because of insufficient mouth movement; 2 = no improvement, but there was sufficient mouth movement; 3 = improved lipreading by producing recognizable sounds; 4 = effective and can speak words; and 5 = very effective and can make sentences [6]

*IQR* interquartile range, *N* not capable of manipulation

^*^The patient, owing to tetraplegia from a spinal cord injury, required assistance to control the on/off switch

Regarding complications, one patient experienced decreased oxygen saturation due to exacerbated coughing during phonation (Table 3). No cardiovascular effects were observed; the device did not interfere with the tracheostomy tube. The vibrations of the device did not affect the triggering of mechanical ventilation without adjusting ventilator settings. Additionally, two patients required minor transducer position adjustments during evaluation.

**Table 3:** Complications associated with the hands-free electrolarynx

Complication	Number of cases	Details
Respiratory	1 (Case 4)	Decreased oxygen saturation due to exacerbated coughing during phonation. The patient required additional time to resume phonation.
Hemodynamic	0	No hemodynamic effects were observed.
Others		
Interference with tracheostomy tube	0	No interference with the tracheostomy tube
Transducer position adjustments needed	2 (case 5, 7)	Minor adjustments to the transducer position were necessary to produce intelligible speech during the speech test.

## Discussion

One of the principal factors contributing to effective communication in critically ill patients with artificial airways is the ability to achieve independent communication [7]. Independent electrolarynx use typically depends on adequate muscle strength to hold and securely position the device. Improper contact with the neck and surrounding tissues may produce radiated noise, potentially compromising speech intelligibility [8]. Sedation level can also influence training effectiveness. Rose et al. [4] reported that user-friendliness and the capacity for independent use facilitated the utilization of the electrolarynx among critically ill patients. Once the hands-free electrolarynx transducer was placed on the patient’s neck, some were able to produce intelligible speech independently, demonstrating their ability to autonomously initiate conversations. Although the EES of the hands-free electrolarynx were comparable to those of the conventional electrolarynx with assistance, the hands-free device may support greater patient independence in communication.

One patient experienced decreased oxygen saturation due to increased coughing during phonation using the hands-free electrolarynx. This patient (Case 4) had the worst oxygenation capacity, characterized by excessive sputum production due to pneumonia. Theoretically, the respiratory cycle was independent during electrolarynx speech because it did not rely on airflow. However, mechanically ventilated patients often either exhaled or stopped breathing temporarily during phonation using the electrolarynx, with most patients using the pressure support mode to regulate the respiratory cycle better. For patients with low respiratory reserve, synchronizing their breathing cycle with speech is particularly challenging. In addition, speech using the electrolarynx requires precise, exaggerated mouth movements and slow articulation for optimal intelligibility, which may further contribute to patient ventilator desynchrony in this patient population [9]. Further research is necessary to explore the link between respiratory status and complications arising from electrolarynx use.

This study has several limitations. First, the small sample size due to the limited number of eligible patients may reduce the generalizability of our findings. Although our findings suggest potential clinical advantages of the hands-free wearable electrolarynx, further large-scale studies are necessary to comprehensively evaluate its effectiveness and broader clinical applicability. Second, the duration of electrolarynx use in this study was short, and the long-term feasibility and safety of the device needed to be evaluated. Third, the devices were not blinded to the raters, which could have introduced bias in the assessment process. Moreover, the experienced research team assisted in using the conventional electrolarynx, which may have influenced the comparison between the devices. The learning curve and user adaptability of the hands-free electrolarynx were not fully evaluated in this study. Further research is needed to quantify the time required for users to become proficient and to assess how usability evolves with experience. Furthermore, the EES evaluation was perceptual and reliant on subjective judgment. Finally, the study was conducted at a single center in Japan with only native Japanese speakers, which may limit the generalizability of our findings to populations speaking other languages or to different clinical settings.

In conclusion, our case series provides preliminary evidence indicating the feasibility of using a hands-free electrolarynx for communication in tracheostomized, mechanically ventilated patients. When used independently by patients, the hands-free electrolarynx achieved greater intelligibility than the conventional electrolarynx. This device may reduce the need for external assistance, potentially enhancing effective communication with medical staff and family members.

## Supplementary Information

Below is the link to the electronic supplementary material.

Supplementary file1 (MP4 39887 KB)

Supplementary file2 (PDF 180 KB)

## Data Availability

The datasets used and analyzed during the current study are available from the corresponding author upon reasonable request.
