# Supplementary material for: A hands-free wearable electrolarynx for communication in tracheostomized mechanically ventilated critically ill patients: a case series
Source: J Anesth. 2025 Mar 12;39(5):810–4. doi: 10.1007/s00540-025-03481-2 (PMC12464148; doi:10.1007/s00540-025-03481-2)
Supplement: Supplementary file 2 — Supplementary file2 (PDF 180 KB) [file 540_2025_3481_MOESM2_ESM.pdf]

Article title: A hands -free wearable electrolarynx for communication in tracheostomized mechanically ventilated critically ill patients: A case series

Journal name: Journal of Anesthesia

Author names: Koji Sato[1]*, Junji Genda[2], Seiki Deguchi[2], and Takumi Taniguchi[1]

Affiliation:
1 Intensive Care Unit, Kanazawa University Hospital, 13-1 Takara-machi, Kanazawa 920-8641, Japan
2 Department of Rehabilitation, Kanazawa University Hospital, 13-1 Takara-machi, Kanazawa 920-8641, Japan.

E-mail address of the corresponding author: rijyuma@yahoo.co.jp (Koji Sato)

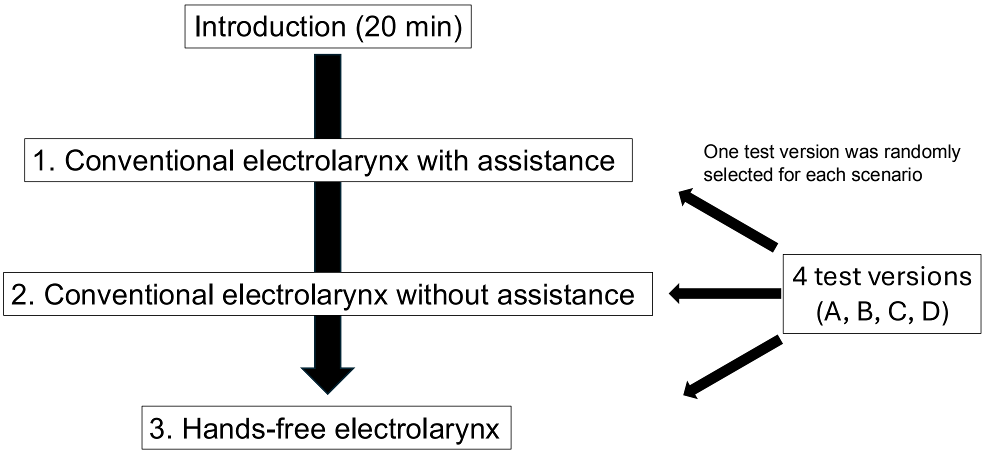

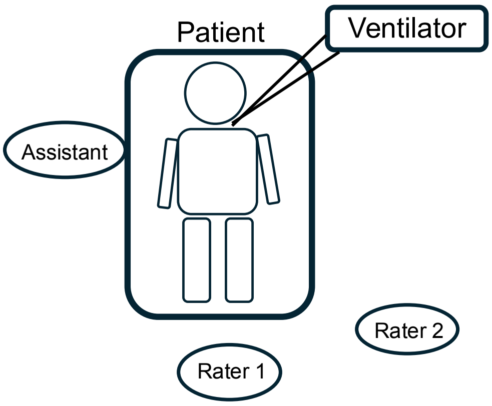

Online Resource 2. Electrolaryngeal speech intelligibility test in tracheostomized patients. After informed consent was obtained, a 20-minute orientation on the electrolarynx was provided. Sedatives and narcotics were meticulously titrated to maintain the patient's level of consciousness to ensure a Richmond Agitation-Sedation Scale (RASS) of 0. Additionally, we assessed whether the patient's lips and tongue had sufficient mobility. An experienced research team member demonstrated the device's operation by positioning it on the participant's neck, focusing on optimal placement and coordinating mouth movements to improve speech production. Participants were then encouraged to use the electrolarynx independently. Throughout the introduction, a speech-language pathologist provided oversight and guidance as necessary. Speech intelligibility was assessed after a brief orientation on the first day of use. Initially, speech produced using a conventional electrolarynx was evaluated, followed by a hands-free electrolarynx. For the conventional electrolarynx, participants were assessed under two conditions: with research team assistance and independently holding the

device to produce speech (Scenario 1: conventional electrolarynx with assistance; Scenario 2: conventional electrolarynx without assistance). For the hands-free electrolarynx (Scenario 3), the research team attached and secured the hands-free electrolarynx transducer to the participant's neck, carefully identifying the most effective placement. The participant was responsible solely for operating the on-off switch. The research team provided participants with words and sentences in large print and instructed them to produce speech using the electrolarynx. Four versions of the speech test (A, B, C, and D) were created, and three versions were randomly assigned to each participant for each scenario. Two raters, seated facing the participant, evaluated the speech. At least one speech-language pathologist participated in the speech intelligibility test as a rater. The effectiveness of electrolarynx speech was graded using the 5-point Electrolarynx Effectivity Score (EES) developed by Tuinman et al. (1 = no improvement in intelligibility; 5 = very effective, capable of producing sentences) [6]. Their scores were then averaged to yield a final EES. Patients with an EES of 4 or higher were classified as effective users.

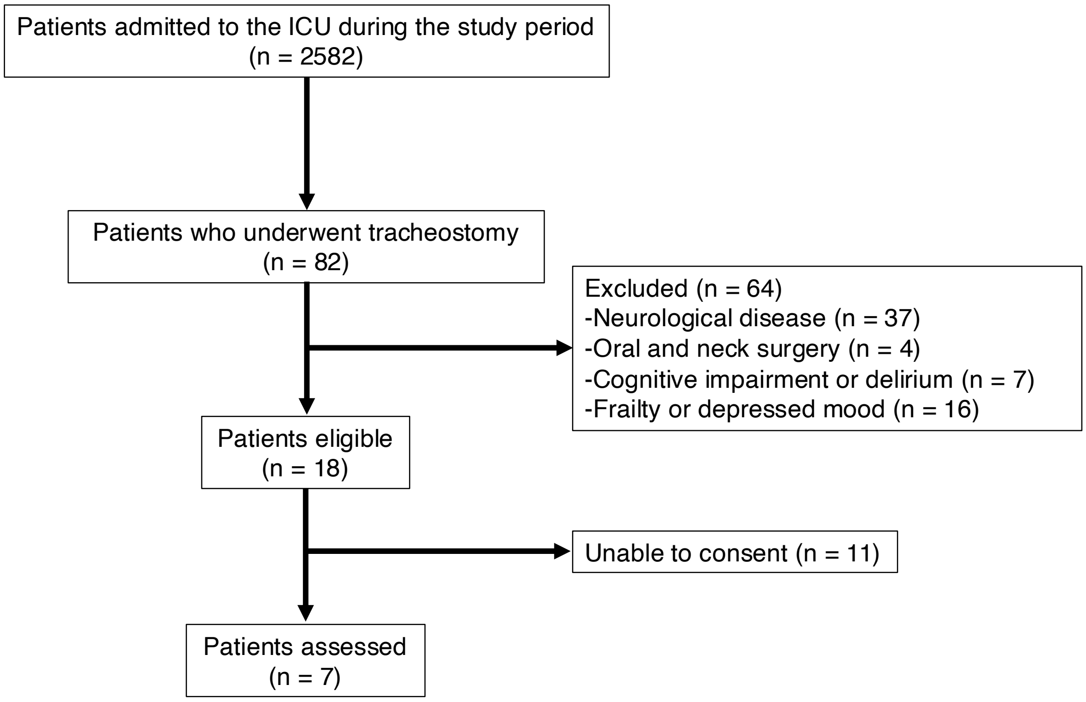

Online Resource 3. Patient enrollment flow diagram. ICU, Intensive care unit.

Case	Richmond Agitation-Sedation Scale (RASS)	Sedative Agent	Narcotic Agent
1	0	None	None
2	0	None	None
3	0	Dexmedetomidine	None
4	0	None	Morphine
5	0	None	Morphine
6	0	None	None
7	0	None	Morphine

Online Resource 4. Sedation levels and administration of sedative and narcotic drugs during Assessment
